# A Bioluminescent Cell Assay to Quantify Prion Protein Dimerization

**DOI:** 10.1038/s41598-018-32581-1

**Published:** 2018-09-21

**Authors:** Katharina Annick Wüsten, Pasham Parameshwar Reddy, Andrej Smiyakin, Maria Eugenia Bernis, Gültekin Tamgüney

**Affiliations:** German Center for Neurodegenerative Diseases (DZNE), Bonn, 53127 Germany

## Abstract

The prion protein (PrP) is a cell surface protein that in disease misfolds and becomes infectious causing Creutzfeldt-Jakob disease in humans, scrapie in sheep, and chronic wasting disease in deer and elk. Little is known regarding the dimerization of PrP and its role in disease. We developed a bioluminescent prion assay (BPA) to quantify PrP dimerization by bimolecular complementation of split *Gaussia* luciferase (GLuc) halves that are each fused to PrP. Fusion constructs between PrP and N- and C-terminal GLuc halves were expressed on the surface of RK13 cells (RK13-DC cells) and dimerized to yield a bioluminescent signal that was decreased in the presence of eight different antibodies to PrP. Dimerization of PrP was independent of divalent cations and was induced under stress. Challenge of RK13-DC cells with seven different prion strains did not lead to detectable infection but was measurable by bioluminescence. Finally, we used BPA to screen a compound library for compounds inhibiting PrP dimerization. One of the most potent compounds to inhibit PrP dimerization was JTC-801, which also inhibited prion replication in RML-infected ScN2a and SMB cells with an EC_50_ of 370 nM and 220 nM, respectively. We show here that BPA is a versatile tool to study prion biology and to identify anti-prion compounds.

## Introduction

The prion protein (PrP^C^) is a natural protein that is predominantly expressed on the outer cell membrane of neurons^1^. The structure of PrP^C^ is well characterized and has been determined by nuclear magnetic resonance (NMR) spectroscopy and X-ray crystallography^2,3^. PrP^C^ has an unstructured, flexible N-terminus followed by a globular domain with three α-helices and little β-sheet structure, and is tethered to the cell surface by a carboxy (C)-terminal GPI anchor^4^. During spontaneous or templated misfolding, PrP^C^ undergoes a conformational transition where it loses all of its α-helical content and adopts mostly a β-sheet structure that is not fully defined yet but likely to consist of a four-rung ß-solenoid architecture^5,6^. This β-sheet-rich conformer, PrP^Sc^, is prone to aggregation, infectious, and toxic to neurons causing neurodegeneration and death^1,7^. Fascinatingly, prion diseases are the only unequivocally confirmed disease group to be sporadic, genetic, and infectious in origin. Prion diseases affect humans and some other mammals, most common in humans being sporadic Creutzfeldt-Jakob disease (sCJD), in cattle bovine spongiform encephalopathy (BSE), in sheep scrapie, and in deer and elk chronic wasting disease (CWD). PrP^Sc^ can exist in multiple conformations — strains — with specific biophysical and biochemical properties that are maintained between hosts upon transmission and determine the clinical manifestation, the phenotype, of a particular prion disease^8^. In humans, for instance, based on the strain, PrP^Sc^ can cause CJD or Kuru, two different human prion diseases with very different incubation times and clinical presentation^9^.

The physiological function of PrP^C^ is not fully understood. A plethora of divergent functions for PrP^C^ have been proposed over the years, leaving it unclear which of them may be more relevant^10,11^. More recent results showing that aged *Prnp* knockout mice develop a chronic demyelinating polyneuropathy^12^ led to the finding that PrP^C^ functions as a ligand to the G protein-coupled receptor Adgrg6 expressed in Schwann cells^13^. Also, identification of PrP^C^ as a member of the ZIP family of metal ion transporters^14^ helped to elucidate its role in polysialylation of neural cell adhesion molecule 1 (NCAM1) during epithelial-to-mesenchymal cell transition^15^. PrP^C^ also has been reported to form homodimers that exist in a monomer-dimer equilibrium, which is a characteristic of receptor proteins involved in signal transduction, and which may also be relevant during the conversion of PrP^C^ to PrP^Sc^^16,17^.

Prion diseases are despite continuing efforts in drug screening to find a treatment, unfortunately, still without cure. Only few drugs have made it into clinical trials, all of which have either failed or are ongoing^18^. Next to transmission experiments to animals many sophisticated tools have been developed over the years to detect and quantify prions and the effect of anti-prion drugs *in vitro*. These include methods as simple as proteolytic treatment of prion-infected cell lysates with proteinase K (PK) to distinguish between PK-sensitive PrP^C^ and PK-resistant PrP^Sc^ after immunodetection with antibodies to PrP. Lately, more refined and scalable methods such as the conformation-dependent immunoassay (CDI)^8^, protein-misfolding cyclic amplification (PMCA)^19^, the standard scrapie cell assay (SSCA)^20^, or the real-time quaking induced conversion assay (RT-QuIC)^21^ have been developed.

To our knowledge no scalable assay has been developed to date to directly study the interaction of prion proteins in cells by bioluminescence. The overall aim of this study was to develop a bioluminescent cell assay that would facilitate studying the biology of the prion protein in its native state. We report here a bioluminescent prion assay (BPA) for the quantification of PrP^C^ dimerization. Based on the concept of protein-fragment complementation^22^ and the fact that PrP^C^ can dimerize, constructs between mouse PrP and split *Gaussia* luciferase halves were expressed in RK13 cells, which were bioluminescent and showed that GPI-anchored fusion constructs of PrP^C^ dimerize on the cell surface under physiological conditions. Treatment of these cells with eight different antibodies to PrP, especially those binding to the first α-helix of PrP^C^, was able to disrupt PrP^C^-mediated dimerization. Dimerization of PrP^C^ fusion constructs did not require divalent cations and was induced under stress when divalent cations were increasingly chelated. Challenge with seven different prion strains of cells expressing PrP^C^ fusion constructs induced bioluminescence within as little as three days. A screen of a library with 1,640 compounds identified 240 compounds inhibiting dimerization of PrP^C^ fusion constructs by 20–85%. JTC-801, a quinoline derivative, potently inhibited dimerization of PrP^C^ fusion constructs by 80% and prion replication in RML-infected ScN2a and SMB cells with an EC_50_ of 370 nM and 220 nM, respectively. Our data shows that the bioluminescent prion assay is a versatile tool to study the biology of prion proteins, and that it can be used to identify compounds inhibiting PrP^C^ dimerization that also inhibit prion replication.

## Results

### Design of fusion constructs between PrP and N- and C-terminal Gaussia luciferase halves

**Figure 1:**
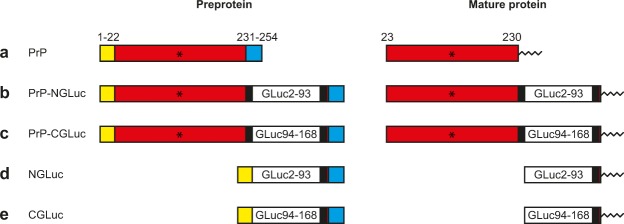
Schematic presentation of PrP-luciferase fusion constructs. (**a**) A signal peptide of 22 amino acids (yellow) directs expression of mouse PrP to the endoplasmic reticulum and is cleaved off of the preprotein. Also, amino acids 231–254 (blue) are cleaved off of the preprotein and replaced by a glycosylphosphatidylinositol (GPI) anchor that is attached to serine 230 and tethers mature mouse PrP (red) to the outer cell membrane. (**b**) PrP-NGLuc is a fusion construct that has the N-terminal half, amino acids 2–93, of *Gaussia* luciferase (GLuc2–93) inserted between amino acids 229 and 230 of mouse PrP, and is flanked by the flexible linker sequence GGGGSGGGGS on each side (black). Mature PrP-NGLuc has a GPI anchor attached to its last amino acid, which is serine 230 of PrP. (**c**) PrP-CGLuc is a fusion construct that has the C-terminal half, amino acids 94–168, of *Gaussia* luciferase (GLuc94–168) inserted between amino acids 229 and 230 of mouse PrP, and is flanked by the flexible linker sequence GGGGSGGGGS on each side (black). Mature PrP-NGLuc has a GPI-anchor attached to its last amino acid, which is serine 230 of PrP. (**d**) NGLuc is a fusion construct that has the N-terminal half, amino acids 2–93, of *Gaussia* luciferase (GLuc2–93) inserted between amino acids 22 and 230 of PrP, and is flanked by the flexible linker sequence GGGGSGGGGS on each side (black). Amino acids 23–229 of PrP are missing in this construct. Mature NGLuc has a GPI-anchor attached to its last amino acid, which is serine 230 of PrP. (**e**) CGLuc is a fusion construct that has the N-terminal half, amino acids 94–168, of *Gaussia* luciferase (GLuc94–168) inserted between amino acids 22 and 230 of PrP, and is flanked by the flexible linker sequence GGGGSGGGGS on each side (black). Amino acids 23–229 of PrP are missing in this construct. Mature CGLuc has a GPI-anchor attached to its last amino acid, which is serine 230 of PrP. All constructs carry the 3F4 epitope (asterics), which allows detection with the 3F4 antibody.

To study dimerization of the prion protein (PrP) by bioluminescence in cells, we cloned fusion constructs between the PrP (Fig. 1a) and the N- and C-terminal halves of split *Gaussia* luciferase (GLuc) into a bicistronic expression vector that allows expression of two proteins from a single mRNA using an internal ribosomal entry site (IRES). Insertion of amino acids 2–93 of GLuc flanked by two flexible linker sequences consisting of GGGGSGGGS between amino acids 229 and 230 of PrP yielded PrP-NGLuc (Fig. 1b), and similar insertion of amino acids 94–168 of GLuc yielded PrP-CGLuc (Fig. 1c). To obtain N- and C-terminal halves of GLuc that are also expressed on the cell surface as PrP-NGLuc and PrP-CGLuc but essentially lack all of PrP that enables dimerization, we inserted N- and C-terminal halves of GLuc with only a C-terminal flexible linker between amino acids 22 and 230 of PrP yielding NGLuc (Fig. 1d) and CGLuc (Fig. 1e). During expression proteins are directed to the endoplasmic reticulum where the leader peptide 1–22 of PrP is cleaved off. Also, the C-terminal signal peptide 231–254 of PrP is replaced by a glycosylphosphatidylinositol (GPI)-anchor attached to serine 230 of PrP, which tethers mature proteins to the outer cell membrane.

### Cellular expression of PrP-NGLuc and PrP-CGLuc results in PrP dimerization and bioluminescence

**Figure 2:**
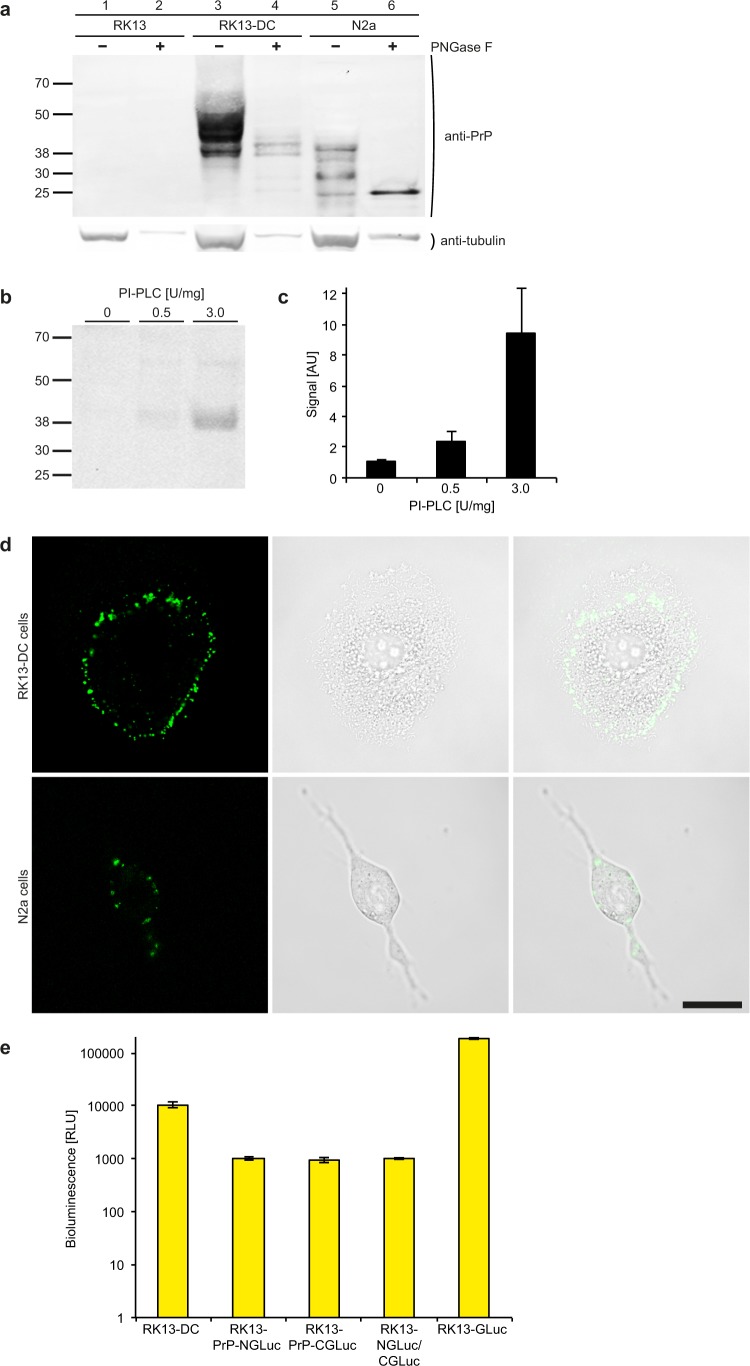
PrP-NGLuc and PrP-CGLuc expression in RK13-DC cells results in bioluminescence. (**a**) Western blot analysis with the Sha31 antibody against PrP shows that RK13-DC cells stably express PrP-NGLuc and PrP-CGLuc from a bicistronic expression vector (lanes 3 and 4). Non-transfected RK13 cells do not express PrP and do not show a detectable signal for PrP (lanes 1 and 2), whereas N2a cells show the characteristic signal for wild-type PrP (lanes 5 and 6). Deglycosylation of cell lysates with peptide-N-glycosidase F (PNGase F, lanes 2, 4, and 6) resulted in lower molecular weight bands suggesting that mature PrP-NGLuc and PrP-CGLuc are properly glycosylated in RK13-DC cells as PrP is in N2a cells. Detection of tubulin on the same blot served as a loading control. Additional lanes were excised for presentation purposes. (**b**) Western blot analysis with the Sha31 antibody against PrP shows that treatment of RK13-DC cells with increasing amounts of phosphoinositide phospholipase C (PI-PLC) increases the amount of detectable protein released into cell culture medium suggesting that mature PrP-NGLuc and PrP-CGLuc are anchored to the outer cell membrane by GPI. Additional lanes were excised for presentation purposes. (**c**) Densitometric quantification of the PrP signal from three different experiments as shown in (**b**). (**d**) Life cell immunofluorescence staining with the Sha31 antibody to PrP (left panels) shows that PrP-NGLuc and PrP-CGLuc are expressed in RK13-DC cells (top row), similar to wild-type PrP in N2a cells (bottom row). Bright-field microscopy (centre panels) shows the contours of the imaged cells. An overlay of fluorescence and bright-field images (right panels) shows that PrP-NGLuc, PrP-CGLuc, and PrP are located at the cell membrane. Bar = 20 µm. (**e**) The bioluminescence measured from RK13-DC cells expressing both PrP-NGLuc and PrP-CGLuc was more than 10-fold above background levels measured from RK13 cells stably expressing only PrP-NGLuc or PrP-CGLuc, or both NGLuc and CGLuc together each lacking the PrP moiety. A functional luciferase consisting of its N- and C-terminal halves was only formed in RK13-DC cells where dimers between the PrP moieties of PrP-NGLuc and PrP-CGLuc could form. In comparison to PrP-NGLuc and PrP-CGLuc in RK13-DC cells, bioluminescence of full-length GLuc in RK13 cells was almost 17-fold higher. In (**c**,**e**) error bars indicate SD.

**Figure 3:**
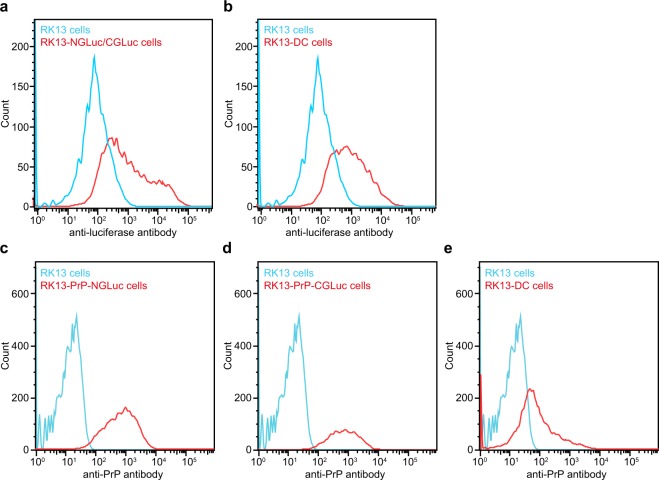
Fusion constructs are stably expressed on the surface of RK13 cells. (**a**) FACS analysis with an antibody to *Gaussia* luciferase confirms that in contrast to non-transfected RK13 cells (blue) RK13-NGLuc/CGLuc cells (red) stably express NGLuc and CGLuc lacking the PrP moiety on the cell surface. (**b**) Equally, the same anti-luciferase antibody also confirms expression of PrP-NGLuc and PrP-CGLuc on the surface of RK13-DC cells (red) that yield a right-shifted signal in comparison to non-transfected RK13 cells (blue). (**c**) FACS analysis with the SAF32 antibody against PrP confirms that in contrast to RK13 cells (blue), stably transfected RK13-PrP-NGLuc cells (red) express PrP-NGLuc on their cell surface. (**d**) In contrast to RK13 cells (blue), stably transfected RK13-PrP-CGLuc cells (red) express PrP-CGLuc on their surface, as determined with SAF32 antibody. (**e**) A signal for PrP was also detected with the SAF32 antibody in RK13-DC cells (red) stably expressing both PrP-NGLuc and PrP-CGLuc but not from non-transfected RK13 cells (blue).

Because the rabbit kidney epithelial cell line RK13 lacks endogenous expression of rabbit PrP (Fig. 2a) as reported previously^23^, we used these cells to stably coexpress PrP-NGLuc and PrP-CGLuc (RK13-DC cells), and other control constructs. Expression of these fusion constructs on the cell surface was confirmed by FACS analysis with an antibody against GLuc (Fig. 3a,b), and the SAF32 antibody against PrP (Fig. 3c–e). Biochemical analysis of cell lysates after western blotting with antibodies to PrP showed that the fusion constructs ran at a higher molecular weight than observed for PrP^C^ in N2a cells. Also, treatment of cell lysates with peptide-N-glycosidase F (PNGaseF) revealed that PrP-NGLuc and PrP-CGLuc were properly glycosylated in RK13 cells because deglycosylation reduced the multiple bands seen without PNGaseF treatment to two major bands representing PrP-NGLuc and the slightly smaller PrP-CGLuc (Fig. 2a and Supplementary Fig. 1A). Biochemical analysis of cell lysates after western blotting with an antibody to GLuc showed that also NGLuc and CGLuc were expressed in RK13-NGLuc/CGLuc control cells and ran at a much lower molecular weight size representative for the lack of PrP in these constructs (Supplementary Fig. 1A). Treatment of RK13-DC cells with increasing amounts of phosphoinositide phospholipase C (PI-PLC) increased the amount of protein detectable in the cell culture supernatant after western blotting with an antibody against PrP suggesting that mature PrP-NGLuc and PrP-CGLuc were anchored to the outer cell membrane by GPI (Fig. 2b,c). Live cell imaging with an antibody to PrP showed that PrP-NGLuc and PrP-CGLuc were expressed on the surface of RK13-DC cells as PrP on N2a cells (Fig. 2d and Supplementary Fig. 1B). Equally, immunocytochemistry with an antibody to GLuc showed that NGLuc and CGLuc were expressed on the surface of RK13-NGLuc/CGLuc cells (Supplementary Fig. 1B). Bioluminescence measurements in RK13-DC cells resulted in a more than 10-fold higher signal in comparison to RK13 cells expressing only PrP-NGLuc or PrP-CGLuc, or NGLuc and CGLuc together (Fig. 2e), suggesting that PrP-NGLuc and PrP-CGLuc dimerize in RK13-DC cells but not NGLuc and CGLuc in RK13-NGLuc/CGLuc negative control cells. In comparison to PrP-NGLuc and PrP-CGLuc in RK13-DC cells, bioluminescence of full-length GLuc in RK13 cells was nearly 17-fold higher (Fig. 2e).

### Dimerization of PrP is inhibited by anti-PrP antibodies

**Figure 4:**
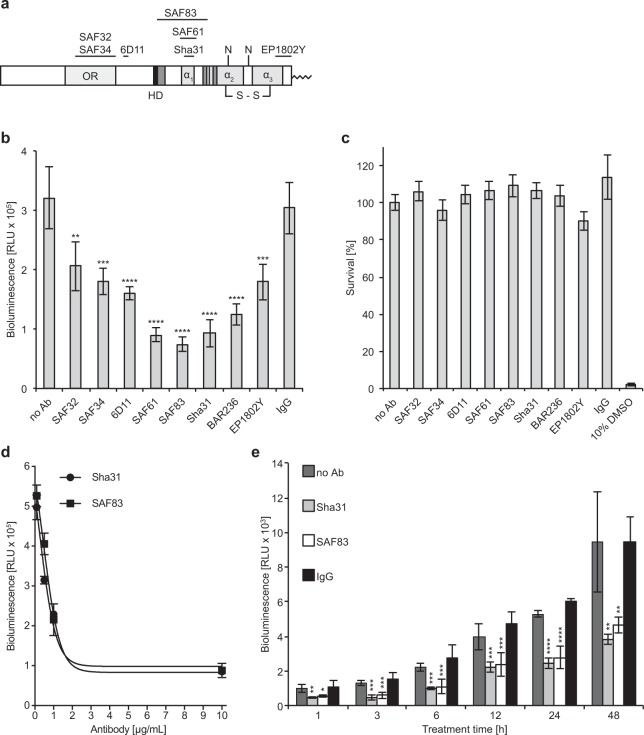
Antibodies to PrP inhibit its dimerization. (**a**) We measured the effect of eight different PrP antibodies on PrP dimerization in RK13-DC cells by bioluminescence assay. SAF32 and SAF34 bind within the octarepeat region (OR), 6D11 between the octarepeat and hydrophobic domain (HD, black), SAF83, SAF61, and Sha31 bind to helix 1 (α_1_), EP1802Y to helix 3 (α_3_) in the C-terminus, and BAR236 an unspecified, conformational epitope of PrP. N = glycosylated asparagine residue, S–S = disulfide bond, dark grey shading = β-sheet structure, light grey shading = α-helical structure. (**b**) Bioluminescence measurements in the absence of antibodies were similar to those in the presence of unspecific mouse IgG, which did not affect bioluminescence and PrP dimerization. In contrast, antibodies binding to or near helix 1 of PrP strongly reduced the bioluminescence and dimerization of RK13-DC cells, whereas those binding to more N- and C-terminal regions of PrP, such as the octarepeat region or helix 3, had a smaller effect on bioluminescence. The effect of Bar236 on bioluminescence was close to those antibodies binding to the core of PrP. (**c**) The metabolic activity of RK13-DC cells after antibody treatment for 24 h was similar to untreated cells or cells treated with unspecific mouse IgG suggesting that the antibody treatment was not toxic to the cells as determined by MTT assay. In contrast, treatment of RK13-DC cells for 24 h with 10% DMSO was toxic and abolished all metabolic activity. (**d**) Treatment of RK13-DC cells for 24 h with increasing antibody concentrations of Sha31 or SAF83 showed a dose dependent effect on the reduction of bioluminescence and PrP dimerization. (**e**) Treatment of RK13-DC cells with the Sha31 or SAF83 antibody had an immediate effect on the reduction of bioluminescence, which was already observable within 1 h after treatment by comparison to untreated cells or cells treated with unspecific mouse IgG. The reduction of bioluminescence by the PrP-specific antibodies relative to the controls remained constant over time for up to 48 h, while the absolute bioluminescence increased due to cell division. Error bars indicate SD (****p < 0.0001, ***p < 0.001, **p < 0.01, *p < 0.05; one-way ANOVA, followed by Dunnett’s multiple comparisons test).

To investigate PrP dimerization in more detail, we measured the effect of eight different antibodies (Table 1) against PrP on the bioluminescence in RK13-DC cells. The antibodies SAF32 and SAF34 bind to the octarepeat region (OR), 6D11 between the OR and the hydrophobic domain (HD), SAF83, SAF61, and Sha31 to the first α-helix (α_1_), EP1802Y to the far C-terminus of the third α-helix (α_3_), and BAR236 to an unidentified conformational epitope (Fig. 4a). In contrast to unspecific mouse IgG, which served as a negative control, all eight antibodies tested against PrP reduced the bioluminescence measured from RK13-DC cells after 24 h, suggesting that steric hindrance of bound antibodies inhibited dimerization of PrP (Fig. 4b). Binding of these antibodies to PrP was not toxic to RK13-DC cells within 24 h as determined by an MTT assay (Fig. 4c). Antibodies binding to α_1_ more potently inhibited dimerization of PrP than those binding to more N- or C-terminal domains of PrP or BAR236 binding to a conformational epitope. Increasing antibody concentrations to PrP led to a concentration-dependent reduction in bioluminescence from RK13-DC cells and reached maximal inhibition between 2–3 µg/mL for SAF83 and Sha31 (Fig. 4d). Inhibition of bioluminescence from RK13-DC cells with antibodies to PrP was immediate and could be measured already after 1 h and remained constant over 48 h relative to untreated cells or cells treated with unspecific mouse IgG while the overall bioluminescence intensities increased over time due to cell division (Fig. 4e).

**Table 1:** PrP antibodies, their epitopes, and EC_50_ values.

Antibody	Epitope	EC_50_ [µg/mL]^*^	Reference
SAF32	59-QPHGGGW(x4)-89	2.5	^51^
SAF34	59-QPHGGGW(x4)-89	1.0	^51^
6D11	97-QWNK-100	0.07	^52, 53^
SAF61	142-GSDYEDRYYREN-153	2.5	^51^
SAF83	126-GYMLGSAMSRPMIHFGNDWE DRYYRENMYRYPNQVYYRP-164	0.25	^51^
Sha31	145-YEDRYYRE-152	0.1	^51^
BAR236	conformational	0.1	^51^
EP1802Y	220-ESQAYYDGRRSS-231	n. a.	^54^

*Effective concentration that eliminates 50% of PrP^Sc^ in cell culture.

### Dimerization of PrP is independent of divalent cations

**Figure 5:**
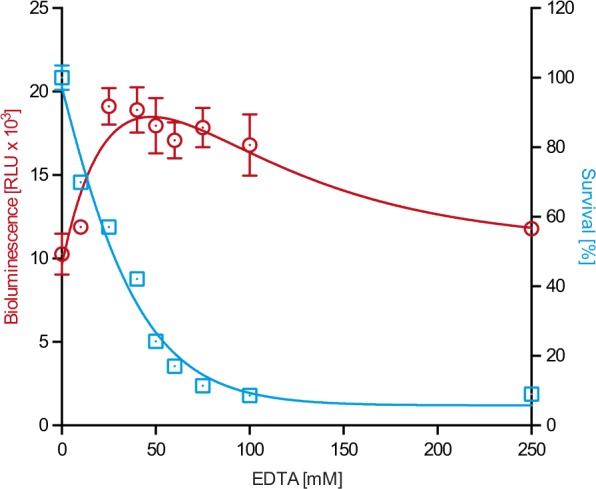
Dimerization of PrP does not require divalent cations. Treatment with increasing concentrations of EDTA for 1 h did not reduce bioluminescence of RK13-DC cells (red circles), suggesting that divalent cations are not required for PrP dimerization. On the contrary, addition of EDTA to RK13-DC cells induced their bioluminescence (red circles), while their metabolic activity (blue squares) was reduced as measured by MTT assay, suggesting that EDTA-induced cell stress promotes PrP dimerization. Error bars indicate SD.

To assess whether dimerization of PrP requires the presence of divalent cations, such as Cu^2+^ or Zn^2+^, we treated RK13-DC cells with increasing concentrations of ethylenediaminetetraacetic acid (EDTA) for 1 h to chelate divalent cations and measured its effect on bioluminescence (Fig. 5). To our surprise chelation of divalent cations did not reduce but induce bioluminescence from RK13-DC cells, suggesting, first, that PrP does not require divalent cations to dimerize, and, second, that dimerization of PrP increases due to EDTA-induced cell stress. Measurement of the metabolic activity of RK13-DC cells by MTT assay showed that increasing concentrations of EDTA were toxic to the cells (Fig. 5).

### BPA detects challenge with different prion strains

**Figure 6:**
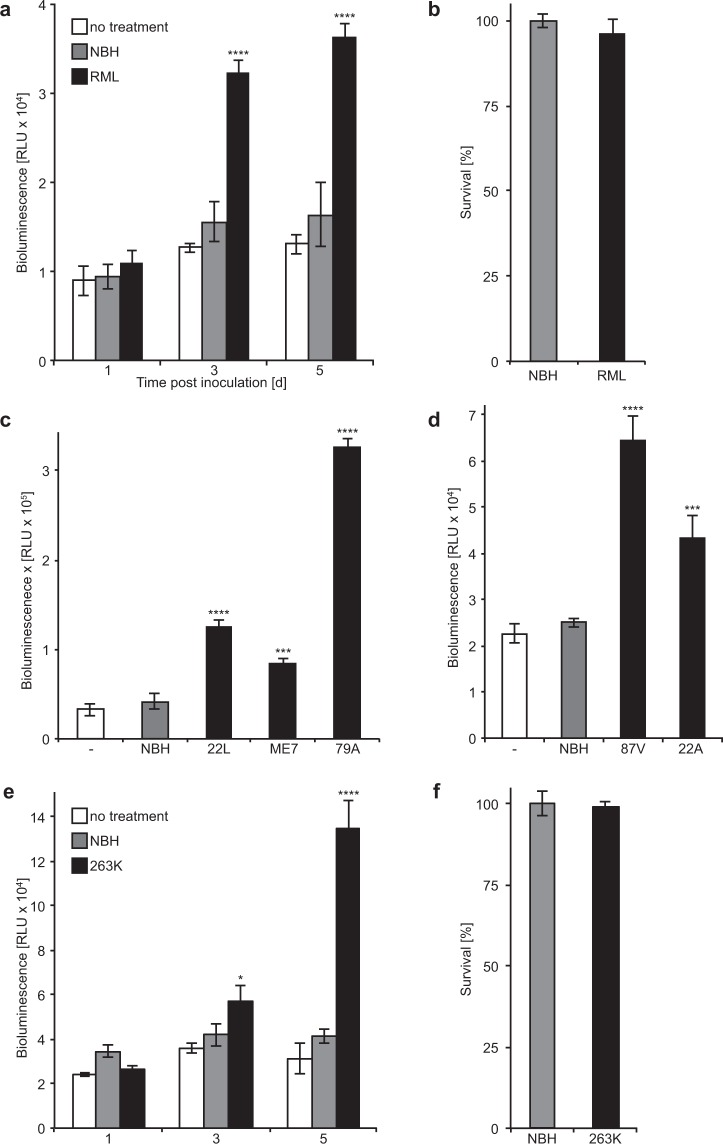
Incubation of RK13-DC cells with prions induces bioluminescence. (**a**) We treated RK13-DC cells for 5 days with normal brain homogenate (NBH) from healthy mice or with brain homogenate from mice that had been sacrificed after they had developed disease due to an infection with RML prions. After one day of treatment the bioluminescence measured from untreated, NBH-treated, and RML-treated RK13-DC cells was comparable, whereas after three days the bioluminescence from RML-treated RK13-DC cells was significantly increased in comparison to the two negative controls. The bioluminescence levels between the three treatment groups did not change much between day three and five. (**b**) Survival of RK13-DC cells treated for 5 days with RML brain homogenate or NBH did not significantly differ between both groups. (**c**) Treatment of RK13-DC cells for five days with brain homogenates from C57BL/6 mice that had developed disease after infection with mouse-adapted scrapie strains 22 L, ME7, and 79 A caused an increase in bioluminescence in comparison to untreated cells or cells treated with NBH. (**d**) Equally, treatment of RK13-DC cells for five days with brain homogenates from VM mice that had developed disease after infection with the mouse-adapted scrapie strains 87 V and 22 A induced bioluminescence in comparison to untreated cells or cells treated with NBH. (**e**) In contrast to RK13-DC cells treated for 5 days with NBH, treatment with hamster brain homogenate containing the hamster-adapted scrapie strain 263 K progressively induced bioluminescence after 3 and 5 days. (**f**) An MTT assay revealed that a 5-day treatment of RK13-DC cells with the hamster-adapted scrapie strain 263 K was not more toxic than treatment with NBH. Error bars represent SD (****p < 0.0001, ***p < 0.001; one-way ANOVA, followed by Dunnett’s multiple comparisons test).

To evaluate whether challenge with prions could be detected by bioluminescence in RK13-DC cells, we challenged the cells with brain homogenate from mice that had developed prion disease after infection with the mouse-adapted scrapie RML prion strain. The bioluminescence of RML-challenged RK13-DC cells was not elevated after one day but significantly increased within three days after challenge and did not further increase after five days in comparison to untreated cells or cells treated with normal brain homogenate (NBH) from healthy mice (Fig. 6a). Survival of RK13-DC cells did not significantly differ between cells treated for 5 days with RML brain homogenate or NBH (Fig. 6b). Next to treatment with RML prions, bioluminescence in RK13-DC cells was also significantly induced after treatment with five additional mouse-adapted scrapie prion strains. These strains comprised 22 L, ME7, and 79 A (Fig. 6c) that similar to RML prions are passaged in C57BL/6 mice expressing *Prnp*-A, and the two prion strains 87 V and 22 A (Fig. 6d) that are passaged in VM mice expressing *Prnp*-B. Surprisingly, challenge of RK13-DC cells with hamster brain homogenate harbouring the hamster-adapted scrapie strain 263 K also lead to an increase in bioluminescence that was not evident after one day but progressively increased from three to five days (Fig. 6e). Similar to challenging RK13-DC cells with RML prions, challenge with 263 K prions did not affect the survival of RK13-DC cells more than NBH (Fig. 6f). To better differentiate between exogenously added PrP^Sc^ in the inoculum and endogenously converted PrP-NGLuc and PrP-CGLuc in RK13-DC cells, we had introduced the 3F4 epitope, which is absent in PrP^Sc^ in the RML brain homogenate, into the PrP moiety of PrP-NGLuc and PrP-CGLuc that allows their specific detection with the 3F4 antibody. When we tested RK13-DC cell lysates after challenge with RML prions by limited digestion with Proteinase K (PK) and western blotting with antibodies against PrP for the presence of PK-resistant PrP^Sc^, we only detected residual PrP^Sc^ from the RML brain homogenate used to infect the cells but not PK-resistant forms of PrP-NGLuc or PrP-CGLuc in RK13-DC cells (Supplementary Fig. 2). Together with the increase in bioluminescence after prion challenge of RK13-DC cells these results suggest that PrP-NGLuc and PrP-CGLuc bind to PrP^Sc^ aggregates but that they are not converted into PK-resistant species.

### Identification of anti-prion compounds

**Figure 7:**
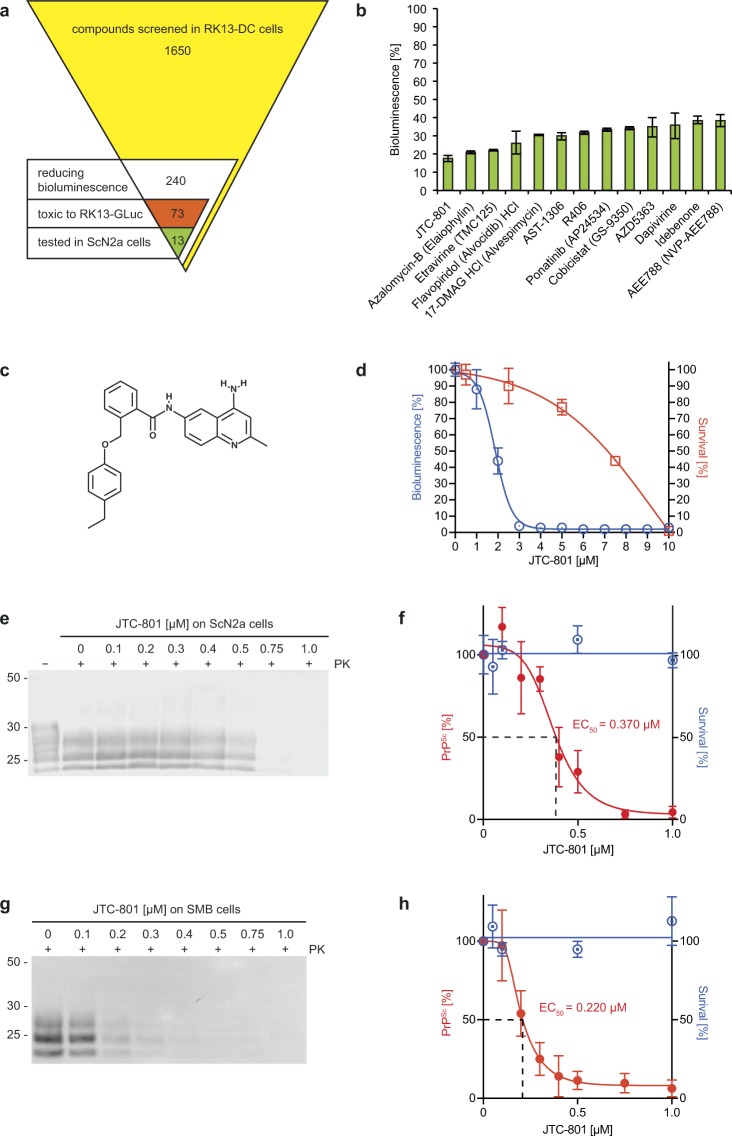
Compound screen identifies small molecules inhibiting PrP dimerization and conversion. (**a**) The Pharmacologically Active Compound Library (Selleckchem) was screened in RK13-DC cells for compounds reducing bioluminescence at a concentration of 10 µM after a 24-hour treatment. From among the 1,650 compounds 240 were found to reduce bioluminescence between 20–85%. From among these 73 compounds were found to reduce bioluminescence in a counter screen in RK13 cells stably expressing full-length *Gaussia* luciferase (RK13-GLuc cells), suggesting that they were either toxic or negatively affecting luciferase activity. From among the remaining 167 compounds, 13 among the most potent to reduce dimerization without negatively affecting bioluminescence of RK13-GLuc cells were selected for further analysis of their anti-prion activity in ScN2a cells. (**b**) The 13 selected compounds strongly reduced bioluminescence of RK13-DC cells by 60–80%. (**c**) From among these 13 compounds the most potent to reduce bioluminescence was JTC-801, a quinoline derivative. (**d**) The EC_50_ for the reduction of bioluminescence of RK13-DC cells for JTC-801 was close to 2 µM, a concentration that did not affect cell survival within 24 h. (**e**,**f**) Biochemical and densitometric analysis shows that a 5-day treatment with JTC-801 reduces the amount of PrP^Sc^ in RML-infected ScN2a cells with an EC_50_ of 370 nM. For presentation purposes additional lanes were excised from the image. Treatment of ScN2a cells with JTC-801 for 5 days did not affect cell for survival for concentrations ≤1.0 µM. (**g**,**h**) Similarly, a 5-day treatment with JTC-801 reduces the amount of PrP^Sc^ in RML-infected SMB cells with an EC_50_ of 220 nM without being toxic for concentrations up to 1 µM. In (**b**,**d**,**f**,**h**) error bars indicate SD.

To identify compounds inhibiting PrP dimerization, we screened Selleckchem’s Pharmacologically Active Compound Library with 1,650 compounds in RK13-DC cells and identified 240 compounds that reduced bioluminescence between 20–85% (Fig. 7a). From among these, 73 compounds reduced bioluminescence in RK13-GLuc cells stably expressing full-length GLuc, suggesting that they were either toxic or affected luciferase activity by other means. From among the remaining 167 compounds we selected 13 among the most potent (Supplementary Fig. 3), which reduced the bioluminescence of RK13-DC cells by 60–80% (Fig. 7b) but not that of RK13-GLuc cells (Supplementary Fig. 4) showing that they were not toxic or interfering with luciferase activity. From among these 13 compounds the quinoline derivative N-(4-amino-2-methylquinolin-6-yl)-2-(4-ethylphenoxymethyl)benzamide (JTC-801) (Fig. 7c) was the most potent to reduce bioluminescence in RK13-DC cells with an EC_50_ of less than 2 µM (Fig. 7d), a concentration that was not toxic to RK13-DC cells. Equally, JTC-801 was the only compound from among the 13 tested (Supplementary Fig. 5) to reduce the amount of PrP^Sc^ in RML-infected ScN2a cells with an EC_50_ of 370 nM (Fig. 7e,f), and without being toxic at these concentrations (Fig. 7f) and without reducing expression of PrP^C^ (Supplementary Fig. 6A,B). Moreover, JTC-801 also reduced the amount of PrP^Sc^ in RML-infected SMB cells with an EC_50_ of 220 nM without being toxic at these concentrations (Fig. 7g,h) and without reducing expression of PrP^C^ (Supplementary Fig. 6C,D). These results show that BPA can identify compounds inhibiting PrP^C^ dimerization, some of which also effectively inhibit the conformational conversion of PrP^C^ that requires interaction between misfolded and native PrP molecules.

## Discussion

Our data show that fusion constructs between PrP^C^ and N- and C-terminal GLuc halves readily dimerize under physiological conditions on the surface of cells implying that dimerization of PrP^C^ is important for its function. Although numerous functions for PrP^C^ have been proposed in the past, the need for PrP^C^ to dimerize to fulfil any of these functions has not been sufficiently addressed^10–12,15^. It has been suggested that dimerization of PrP^C^ is mediated by its internal hydrophobic domain, without which dimerization and subsequent stress-induced signalling is not possible^24^. Here antibodies (SAF83, SAF61, and Sha31) binding proximally to the hydrophobic domain of PrP sterically hindered dimerization greater than those binding to more distal N- and C-terminal regions of PrP (SAF32, SAF34, and EP1802Y), supporting the concept that the hydrophobic domain of PrP^C^ may be important for dimer formation. Some studies report that PrP-binding antibodies such as SAF61 induce signal transduction by crosslinking PrP molecules^25^. We did not study signal transduction in our cell system but our data shows that antibodies binding near the hydrophobic domain of PrP^C^ potently inhibit its dimerization. This does not necessarily mean that antibodies inhibiting the dimerization of PrP^C^ abolish PrP^C^-mediated signal transduction. In lipid rafts PrP^C^ has also been shown to interact with transmembrane proteins such as NCAM1 to mediate cell-signalling^26,27^, a pathway, which is probably less active when PrP^C^ is sequestered in its dimeric form. By inhibiting dimerization, antibodies such as SAF61 could increase the number of monomeric PrP^C^ molecules that are free to bind NCAM1 and thus induce an alternative signalling pathway^26^, which remains to be shown.

The highly unstructured and conserved N-terminus of PrP^C^ has an octarepeat region with four tandem repeats of PHGGGWGQ and two additional histidines that together can bind 5–6 divalent cations such as Cu^2+^ and Zn^2+^^28,29^. Using BPA we tested whether divalent cations affect dimerization of PrP^C^ under physiological conditions. The concentration of divalent cations in 10% FBS-supplemented DMEM cell culture medium is in the low millimolar range with Ca^2+^ (2.93 mM) and Mg^2+^ (0.93 mM) being the major constituents, and with Zn^2+^ (4.50 µM) and Cu(II)^2+^ (0.34 µM) present only at micromolar concentrations^30^. Interestingly, EDTA concentrations greatly exceeding those of divalent cations did not reduce the bioluminescence of RK13-DC cells showing that PrP^C^ does not require Cu^2+^ or Zn^2+^ or any other divalent cations to dimerize. Surprisingly, the bioluminescence measured from RK13-DC cells was induced in the presence of increasing EDTA concentrations, which were toxic to RK13-DC cells, suggesting that PrP^C^ dimerizes as a cellular response to certain types of stress. Dimerization of PrP^C^ has been reported to be protective against PrP^Sc^-induced cell stress and to require the hydrophobic domain and GPI anchor to be protective^24^. Because the EDTA-induced increase in bioluminescence was measured within 1 h of treatment, stress-induced PrP^C^ dimerization cannot be attributed to *de novo* protein synthesis resulting in increased protein levels but must occur through increased dimerization of monomeric PrP^C^ molecules that are already present. PrP^C^ has been reported to preferentially localize to lipid rafts due to its GPI anchor^31,32^ but PrP^C^ is also present in non-lipid raft membrane regions, and enhanced recruitment of monomeric PrP^C^ to lipid rafts during stress could favour its dimerization and PrP^C^-dependent cell signalling^33^.

Using BPA we could detect challenge of RK13-DC cells with six mouse-adapted and one hamster-adapted sheep scrapie strain. Very few cell lines are susceptible to infection with prions and often the spectrum of strains resulting in detectable infection for each susceptible cell line is very limited^34–38^. Our observations suggest that infected brain homogenate induces dimerization of PrP, which requires three days to become detectable by bioluminescence under these experimental conditions. To complement each other and for bioluminescence to occur, the luciferase moieties of PrP-NGLuc and PrP-CGLuc have to remain in their original conformation after binding to PrP^Sc^. This is possible because both GLuc halves in PrP-NGLuc and PrP-CGLuc are separated from the PrP moiety by a linker domain with 10 amino acids. The extent of conformational change the PrP moiety of PrP-NGLuc and PrP-CGLuc undergoes after binding to PrP^Sc^ remains to be elucidated. Failure to detect a signal for PrP with the 3F4 antibody after proteolytic digestion of prion-challenged RK13-DC cell lysates indicates that PrP-NGLuc and PrP-CGLuc are not converted to a PK-resistant form. Surprisingly, BPA was also able to detect challenge with the hamster-adapted scrapie strain 263 K, which does not cause clinical disease in mice^39^. Interestingly, both hamster-adapted scrapie strains 263 K and Sc237 have been reported to cause subclinical disease in mice, without obvious generation of PK-resistant PrP^Sc^ in the subclinically infected mice. Upon passaging of brain homogenates from these subclinically infected mice, both strains resulted in clinical disease in hamsters and mice^40,41^. Although, NBH did not induce bioluminescence, and both, normal and infected brain homogenates, did not differ in their influence on the survival of RK13-DC cells, we cannot rule out that a component other than PrP^Sc^ from infected brains induces dimerization of PrP-NGLuc and PrP-CGLuc in RK13-DC cells.

Past efforts to identify compounds effectively inhibiting the conversion of PrP^C^ to PrP^Sc^ *in vivo* have proven difficult for several reasons, particularly because the mechanism of conversion and the structure of PrP^Sc^ are poorly understood^18,42^. With a few exceptions most drug screening approaches are ‘black box’ approaches where compounds are screened for their potency to reduce PrP^Sc^ levels in prion-infected cells without knowing their mechanism of action^43^. Because PrP^Sc^ needs to bind to PrP^C^ to convert it, our paradigm was to identify compounds that strongly bind to PrP^C^ and thus inhibit its binding and conversion to PrP^Sc^. Because compounds inhibiting PrP^C^ dimerization are likely to have a strong affinity for PrP^C^, potentially inhibiting its conversion to PrP^Sc^, we screened a small compound library using BPA and identified several compounds that potently inhibited dimerization of PrP^C^ fusion constructs.

Here we have identified JTC-801 as a potent small-molecule inhibitor of prion replication. JTC-801 was first reported as a small-molecule nociceptin antagonist that binds to the nociceptin/orphanin FQ receptor (NOP) also known as the kappa-type 3 opioid receptor^44^. Studies in acute pain animal models revealed that it is an efficacious and potent anti-nociceptive that is available by intravenous injection and oral administration and as such may represent a novel class of analgesics^45^. With an EC_50_ of 370 nM in ScN2a cells and of 220 nM in SMB cells, JTC-801 has a comparable anti-prion efficacy to other quinoline derivatives such as quinacrine with an EC_50_ of 300–400 nM^46,47^. The fact that JTC-801 is an orally available drug and in contrast to quinacrine penetrates the brain in high enough concentrations to be potentially effective against prions *in vivo*, and has also been tested in Phase I and II clinical trials in Japan as an analgesic makes it a promising anti-prion drug to be tested in animal models of prion disease^48,49^.

In summary, the BPA is a novel scalable cell assay to quantify PrP dimerization in dependence of various stimuli. The biology of PrP is poorly understood and being able to quantify PrP dimerization as a response to stress or other stimuli could help to uncover yet unknown functions of PrP. Equally, we have shown here that BPA is a useful tool to identify novel anti-prion compounds like JTC-801 that interfere with PrP^C^ dimerization and its conversion to PrP^Sc^. For the scarcity of human cell lines infected with human prions, many screens utilize mouse cell lines expressing mouse PrP^C^ and infected with mouse-adapted prions to identify anti-prion compounds that later fail to inhibit human prions from replicating when tested in mice or patients expressing human PrP^C^ infected with CJD^50^. Replacement of the mouse PrP sequence in PrP-NGLuc and PrP-CGLuc with species-specific PrP, i.e. human PrP^C^, could greatly improve future efforts to identify species-specific anti-prion compounds.

## Methods

### Cloning of DNA constructs

DNA for humanized *Gaussia princeps* luciferase (GLuc), and fusion constructs between mouse *Prnp* and the N-terminal half of GLuc (PrP-NGLuc), *Prnp* and the C-terminal half of GLuc (PrP-CGLuc), and both constructs giving rise to NGLuc and CGLuc was custom synthesized (Thermo Fisher Scientific). The *Prnp* sequence included the 3F4 epitope with leucine 108 and valine 111 both mutated to methionine. The start codon of PrP-NGLuc and NGLuc was preceded by the Kozak sequence GCCACC. DNA sequences encoding amino acids 2–93 or 94–168 of GLuc were inserted between *Prnp* nucleotides encoding amino acids 229 and 230 of PrP and were flanked by sequences encoding a flexible GGGGSGGGS linker on both sides. To obtain the negative control constructs NGLuc and CGLuc, DNA sequences encoding amino acids 2–93 or 94–168 of GLuc with only a C-terminal GGGGSGGGS linker were inserted between *Prnp* nucleotides encoding amino acids 22 and 230 of PrP, eliminating most of the PrP sequence except for its N- and C-terminal signal peptides. PrP-NGLuc and PrP-CGLuc together, and NGLuc and CGLuc together were cloned into a modified pIRES2-DsRed-Express vector that allows bicistronic expression via an internal ribosomal entry site (IRES) element from a single mRNA. PrP-NGLuc and NGLuc were inserted via an N-terminal *Nhe*I and a C-terminal *Eco*RI restriction site, and PrP-CGLuc and CGLuc via an N-terminal *Pml*I and a C-terminal *Xho*I restriction site. GLuc was cloned into the pIRES2-DsRed-Express vector via an N-terminal *Nhe*I and a C-terminal *Xho*I restriction site eliminating the IRES element. All constructs were confirmed for correctness by DNA sequencing (GATC Biotech).

### Cell culture

RK13 cells (American Type Culture Collection), a rabbit kidney epithelial cell line, and N2a cells (American Type Culture Collection), a mouse neuroblastoma cell line, and ScN2a and SMB cells infected with the mouse-adapted scrapie strain RML (kindly provided by Dr. David Westaway and Dr. Debbie McKenzie, University of Alberta) were grown in DMEM high glucose medium (Thermo Fisher Scientific) supplemented with 10% (v/v) foetal bovine serum (FBS; Thermo Fisher Scientific) and 1% (v/v) penicillin and streptomycin (Sigma) and incubated at 37 °C and 5% CO_2_. Medium was exchanged every third to fourth day. For transfections of RK13 cells, Lipofectamine 2000 (Thermo Fisher Scientific) was used following the manufacturer’s instructions. Transfected RK13 cells were analysed by fluorescence activated cell sorting (FACS) with the SAF32 antibody against PrP (Bertin Pharma) or the anti-GLuc antibody (New England Biolabs) against *Gaussia* luciferase for expression of PrP-NGLuc and PrP-CGLuc and similar constructs at the Flow Cytometry Core Facility of the University of Bonn using a BD FACSAria III (Becton Dickinson). RK13 cells expressing high surface protein levels were FACS sorted and maintained in 700 μg/mL G418 (Sigma) to obtain stably expressing RK13 cell lines.

### Live cell imaging

For live cell imaging, N2a and RK13-DC cells were cultured in DMEM high glucose medium (Thermo Fisher Scientific) supplemented with 10% (v/v) foetal bovine serum (FBS; Thermo Fisher Scientific) and 1% (v/v) penicillin and streptomycin (Sigma) in a 35 mm high µ-Dish (Ibidi) and then incubated with the SAF32 antibody against PrP for 1 h at 37 °C. After three washes with PBS (pH 7.4), cells were incubated for 1 h with a secondary goat anti-mouse antibody conjugated to Alexa Fluor 488 (Thermo Fisher Scientific) followed by three washes with medium. Live images were visualized and recorded using an LSM700 confocal microscope (Carl Zeiss).

### Survival Assay

Five thousand ScN2a cells or 10,000 stably transfected RK13 cells per well were cultured in flat-bottomed 96-well plates in DMEM medium without phenol red (Thermo Fisher Scientific) containing 10% FBS and 1% penicillin and streptomycin. After 6 h the cells were treated either with antibodies or selected compounds. Treatment with 10% dimethyl sulfoxide (DMSO; Sigma) served as a positive control for cell toxicity. After 5 days of incubation cell viability was measured in three replicates using the MTT Cell Proliferation Assay Kit (Trevigen) according to the manufacturer’s instructions. Briefly, after addition of 10 μL 3-(4,5-dimethylthiazol-2-yl)-2,5-diphenyltetrazolium bromide (MTT) reagent to each well, the cells were incubated for 3 h at 37 °C. Afterwards 100 μL detergent reagent was added to each well and the plates were incubated for another 2 h at 37 °C to stop the reaction, to lyse the cells, and to solubilize the formazan dye. Absorbance was measured at 570 nm from each well using a Fluostar Omega microplate reader (BMG Labtech) with a reference wavelength set to 690 nm.

### Biochemical analysis

To prepare cell lysates for biochemical analysis, cells were lysed for 5 to 10 min in lysis buffer containing 10 mM Tris-HCl (pH 7.4), 150 mM NaCl, 0.5% (w/v) deoxycholate, and 0.5% (v/v) Triton X-100. Lysates were centrifuged at 1,500 x g for 5 min at 4 °C to remove cell debris. The protein concentration of supernatants was measured with the Pierce BCA Protein Assay Kit (Thermo Fisher Scientific). To detect Proteinase K (PK)-resistant PrP, cell lysates and brain homogenates were incubated with PK (New England Biolabs) at an enzyme to protein ration of 1:50 for 1 h at 37 °C with shaking at 300 rpm. PK activity was quenched by adding PMSF to a final concentration of 2 mM. To detect proper glycosylation, samples were treated with Peptide N-Glycosidase F (PNGaseF; New England Biolabs) according to the manufacturer’s instructions. Samples were further centrifuged at 100,000 × g for 1 h at 4 °C and pellets dissolved in lysis buffer and NuPAGE LDS sample buffer, boiled for 10 min, and loaded onto 4–12% NuPAGE Bis-Tris gels (Thermo Fisher Scientific). SDS-PAGE (sodium dodecyl sulfate-polyacrylamide gel electrophoresis) was processed in a MOPS buffer system (Thermo Fisher Scientific). Gels were blotted to PVDF membranes (Merck Millipore), blocked with 5% (w/v) milk in Tris-buffered saline supplemented with 0.05% Tween 20 for 1 h at room temperature and then probed with the Sha31 (Bertin Pharma) or 3F4 (BioLegend) antibody against PrP, or the anti-GLuc (New England Biolabs) antibody against *Gaussia* luciferase at a 1:2,000 dilution and an antibody against β-tubulin (Thermo Fisher Scientific) at a 1:2,000 dilution over night at 4 °C. Blots were developed with IRDye 680- or IRDye 800-conjugated goat anti-mouse or goat anti-rabbit secondary antibodies (LI-COR Biosciences), and imaged with an Odyssey infrared imaging system (LI-COR Biosciences). Image cropping was done in Adobe Photoshop CS5.1. For release of GPI-linked proteins from the cell surface, approximately 0.5–1.0 × 10^6^ cells were cultured in 60 mm diameter dishes, rinsed twice with cold PBS, and then treated with 0, 0.5, or 3.0 units of phosphoinositide phospholipase C (PI-PLC) from *B*. *cereus* (Thermo Fisher Scientific) in 1.5 mL of PBS for 2 h at 4 °C. Bovine albumin from the recovered buffer was removed by overnight incubation with anti-bovine albumin (BSA)-agarose antibody (Sigma) and subsequent centrifugation at 14,000 × g for 2 min at 4 °C. The recovered supernatant was concentrated to 50 µL (20-fold) using an Amicon Ultra 0.5 mL centrifugal filter with a 3 kDa pore size. For analysis of released proteins, 32.5 µL of concentrated sample was analyzed on a 4–12% NuPAGE Bis-Tris gel (Thermo Fisher Scientific) as described above.

### Bioluminescence assay and compound screening

For bioluminescence measurements, 5,000 stably transfected RK13 cells were plated in 100 µL per well in 96-well half-area plates in DMEM without phenol red containing 10% FBS, 1% penicillin and streptomycin, and 700 μg/mL G418. Depending on the experiment, cells were cultured in the presence of antibodies, brain homogenate, or ethylenediaminetetraacetic acid (EDTA; Sigma) for up to 5 days at 37 °C and 5% CO_2_. Immediately before starting the assay a 1.2 mM stock solution of coelenterazine (Biosynth) in methanol (Sigma) was diluted to a final concentration of 40 μM in DMEM/F-12 without phenol red. Bioluminescence was measured from each well 1 s after 30 µL of diluted coelenterazine was added with an automated pump to a well with a Fluostar Omega microplate reader (BMG Labtech), or after 10 s when the Envision Multilabel platereader (PerkinElmer) was used. Wells without cells served as blanks. Antibodies against PrP used in the bioluminescence assay were reconstituted or diluted in water and used without further purification. All monoclonal PrP antibodies from Bertin Pharma, SAF32, SAF34, SAF61, SAF83, Sha31, and BAR236, were provided as lyophilized IgG with BSA. The monoclonal 6D11 antibody against PrP was produced in the Integra flask system using serum free media and was purified using protein A affinity chromatography and provided in PBS (Covance). The monoclonal EP1802Y antibody against PrP was obtained in 50 mM Tris-Glycine (pH 7.4), 0.15 M NaCl, 40% Glycerol, 0.01% sodium azide and 0.05% BSA (Genetex). Unspecific mouse IgG antibodies were obtained as lyophil from 0.01 M phosphate buffer, pH 7.2, with 0.015 M NaCl after by fractionation and ion-exchange chromatography of pooled normal mouse serum (Sigma). Unless otherwise stated antibodies were used at 1 µg/mL.

For compound screening, 1,650 compounds of the Pharmacologically Active Compound Library (Selleckchem) were screened in triplicates in RK13-DC cells. Screening was carried out in 384-well plates (Nunc) with 5,000 cells per well in 35 µL DMEM without phenol red containing 10% FBS, 1% penicillin and streptomycin, 700 μg/mL G418, and 10 μM compound. Compounds reducing bioluminescence in RK13-DC cells were counter screened in RK13 cells stably expressing full-length *Gaussia* luciferase to identify toxic compounds and those negatively affecting luciferase activity. After 24 h of incubation the bioluminescence was measured from each well after adding 45 µL of 40 µM coelenterazine in DMEM without phenol red using an Envision Multilabel plate reader (PekinElmer). Wells with untreated cells were used as positive controls and wells without cells as blanks.

### Challenge of RK13-DC cells with prions

To expose RK13-DC cells to infectious inocula, 5,000 cells were plated per well in a 96-well half-area plate in 97.5 µL DMEM without phenol red (Thermo Fisher Scientific) containing 10% FBS, 1% penicillin and streptomycin, and 700 μg/mL G418. Brain tissue samples from healthy C57BL6/J mice (NBH, The Jackson Laboratory) and RML-infected C57BL6/J mice (kindly provided by Dr. Walker Jackson, German Center for Neurodegenerative Diseases) were homogenized in Ca^2+^- and Mg^2+^-free phosphate-buffered saline (PBS) by three 60-s-cycles in a Precellys 24-Dual homogenizer (Peqlab) to obtain 10% (w/v) brain homogenates. The mouse-adapted scrapie strains 22 L, ME7, 79 A, 87 V, and 22 A were obtained as 10% (w/v) brain homogenates in PBS (kindly provided by Dr. Jean Manson, the Roslin TSE Resource Centre, University of Edinburgh). The 263 K hamster prion strain was obtained as a 10% (w/v) hamster brain homogenate in PBS (kindly provided by Dr. Walker Jackson). Brain homogenates were centrifuged at 1,500 x g for 5 min at 4 °C to remove cell debris. Before plating the cells, 2.5 µL of a 10% (w/v) brain homogenate in PBS from healthy mice or animals infected either with RML, 87 V, 22 A, 22 L, ME7, 79 A, or 263 K prions was added to each well. Bioluminescence of cells exposed to RML or 263 K prions was measured after 1, 3, and 5 days of incubation, while bioluminescence of cells exposed to other prion strains was measured only after 5 days. Statistical significance was assessed by one-way ANOVA, followed by Dunnett’s multiple comparisons test. All experiments were conducted in accordance with legal regulations in Germany and all experimental protocols were approved by the DZNE’s Committee for Animal Experiments.

## Electronic supplementary material

Supplementary Information
