# Supplementary material for: A Bioluminescent Cell Assay to Quantify Prion Protein Dimerization
Source: Sci Rep. 2018 Sep 21;8:14178. doi: 10.1038/s41598-018-32581-1 (PMC6155003; doi:10.1038/s41598-018-32581-1)
Supplement: Supplementary file 1 — Supplementary Information [file 41598_2018_32581_MOESM1_ESM.pdf]

A Bioluminescent Cell Assay to Quantify Prion Protein Dimerization

Katharina Annick Wüsten[1], Pasham Parameshwar Reddy[1], Andrej Smiyakin[1], Maria Eugenia Bernis[1], Gültekin Tamgüney[1*]

[1] German Center for Neurodegenerative Diseases (DZNE), Bonn, 53127, Germany

* Corresponding author erdem@dzne.de (G.T.)

Supplementary information

Methods

Immunocytochemistry. For cell staining, cells were fixed with 4 % (w/v) paraformaldehyde (PFA, Roth) and blocked with 5 % (v/v) normal goat serum and 2 % (w/v) bovine serum albumin (BSA, Sigma) for 1 h, and then incubated with the SAF32 antibody against PrP overnight at 4 °C. After washing three times with Ca^{2+}- and Mg^{2+}-free PBS (pH 7.4), cells were incubated with a secondary goat anti-mouse antibody conjugated to Alexa Fluor 488 (Thermo Fisher Scientific) for 1 h. Additionally, cells were stained with DAPI (4',6-diamidino-2-phenylindole; Thermo Fisher Scientific), coverslipped with Fluoromount (Sigma), and visualized using an LSM700 confocal microscope (Carl Zeiss).

Supplementary Figure 1. Immunocytochemistry of RK13 cells stably expressing control constructs. (A) Western blot analysis with the Sha31 antibody against PrP shows stable expression from a bicistronic expression vector of PrP-NGLuc and PrP-CGLuc in RK13-DC cells (lanes 1 and 2), of PrP-NGLuc in RK13-PrP-NGLuc cells (lanes 3 and 4), of PrP-CGLuc in RK13-PrP-CGLuc cells (lanes 5 and 6), of NGLuc and CGLuc in RK13-NGLuc/CGLuc cells (lanes 7 and 8, centre panel), and of full-length GLuc in RK13-GLuc cells (lane 9, centre panel). The western blot in the top panel was probed with the Sha31 antibody against PrP, whereas the western blot in the centre panel was probed with an antibody to *Gaussia* luciferase. The blot in the bottom panel was probed with an antibody against tubulin to control for loading. Deglycosylation of cell lysates with peptide-N-glycosidase F (PNGase F, lanes 2, 4, 6, and 8) resulted in lower molecular

weight bands suggesting that mature PrP-NGLuc and PrP-CGLuc are properly glycosylated. PNGaseF treatment of RK13-NGLuc/CGLuc cells did not result in a shift in molecular weight of NGLuc and CGLuc because they are not glycosylated (lane 8 versus 7, centre panel). Bands originate from the same western blot (same acquisition settings). Additional lanes were excised for presentation purposes. (B) Immunofluorescence staining with the Sha31 antibody to PrP (green) shows expression of PrP-NGLuc on the surface of RK13-PrP-NGLuc cells (left panel), of PrP-CGLuc on the surface of RK13-PrP-CGLuc cells (centre panel), and of NGLuc and CGLuc on the surface of RK13-NGLuc/CGLuc cells (right panel). Nuclei are stained with DAPI in blue. Molecular weight is shown in kilodalton. Bar = 20 μm.

Supplementary Figure 2. PrP-NGLuc and PrP-CGLuc are not converted to PK-resistant forms in RK13-DC cells. Biochemical analysis of RML-infected RK13-DC cells shows that PrP-NGLuc and PrP-CGLuc are not converted to a PK-resistant form. When probed with the Sha31 antibody against PrP, RML-infected RK13-DC cells (lane 1 and 2) show a PK-resistant signal after PK digest (lane 2). This signal represents residual inoculum from the RML brain homogenate used to infect the cells. When the same blot was probed with the 3F4 antibody against PrP using the same acquisition settings (lanes 3 and 4) that only recognizes the 3F4 epitope expressed in PrP-NGLuc and PrP-CGLuc and which is absent in PrP^{Sc} from mouse brain, no signal for PrP could be detected after PK digest on the overexposed blot (lane 4). Additional lanes were excised for presentation purposes.

Supplementary Figure 3. Compounds screened in ScN2a cells. Shown are the structures of the 13 compounds that were tested in ScN2a cells for their efficacy to clear prion infection. Each compound is listed with its trivial name, CAS registry number, molecular weight (MW) in [g/mol], and molecular formula (MF).

Supplementary Figure 4. The bioluminescence of RK13-GLuc cells is unaffected by 13 compounds. The bioluminescence of RK13 cells stably expressing full-length GLuc (RK13-GLuc cells) was not affected by the 13 compounds that were selected for further analysis in ScN2a cells during a 24 h-treatment at 10 μM. Shown is the percentage of cellular bioluminescence relative to untreated cells for each compound. Error bars indicate SD.

Supplementary Figure 5. Evaluation of the toxicity and anti-prion activity of the selected compounds. (A) The selected compounds were first tested for their toxicity after five days of treatment, and (B) then evaluated for their anti-prion activity in ScN2a cells after a 5-day treatment at non-toxic concentrations. Additional lanes were excised for presentation purposes. The letters QA stand for quinacrine. Molecular weight is shown in kilodalton. (C) The residual PrP^{Sc} signal was quantified by densitometry from three western blots as shown in B) and is shown as the percentage relative to untreated cells. The letters QA stand for quinacrine. Error bars indicate SD.

Supplementary Figure 6. JTC-801 does not reduce expression of PrP^{C} in ScN2a and SMB cells at concentrations inhibiting prion replication. Western blot analysis of (A)

ScN2a cell lysates or (C) SMB cell lysates with the Sha31 antibody against PrP shows that treatment of these cells with increasing concentrations of JTC-801 for 5 days did not reduce PrP^C expression. Detection of GAPDH on the same blots served as a loading control. Additional lanes were excised for presentation purposes. Molecular weight is shown in kilodalton. (B and D) Expression of PrP and GAPDH was quantified by densitometry from three western blots as shown in A) and C), respectively, and the ratio of both is shown as the mean. Error bars indicate SD.

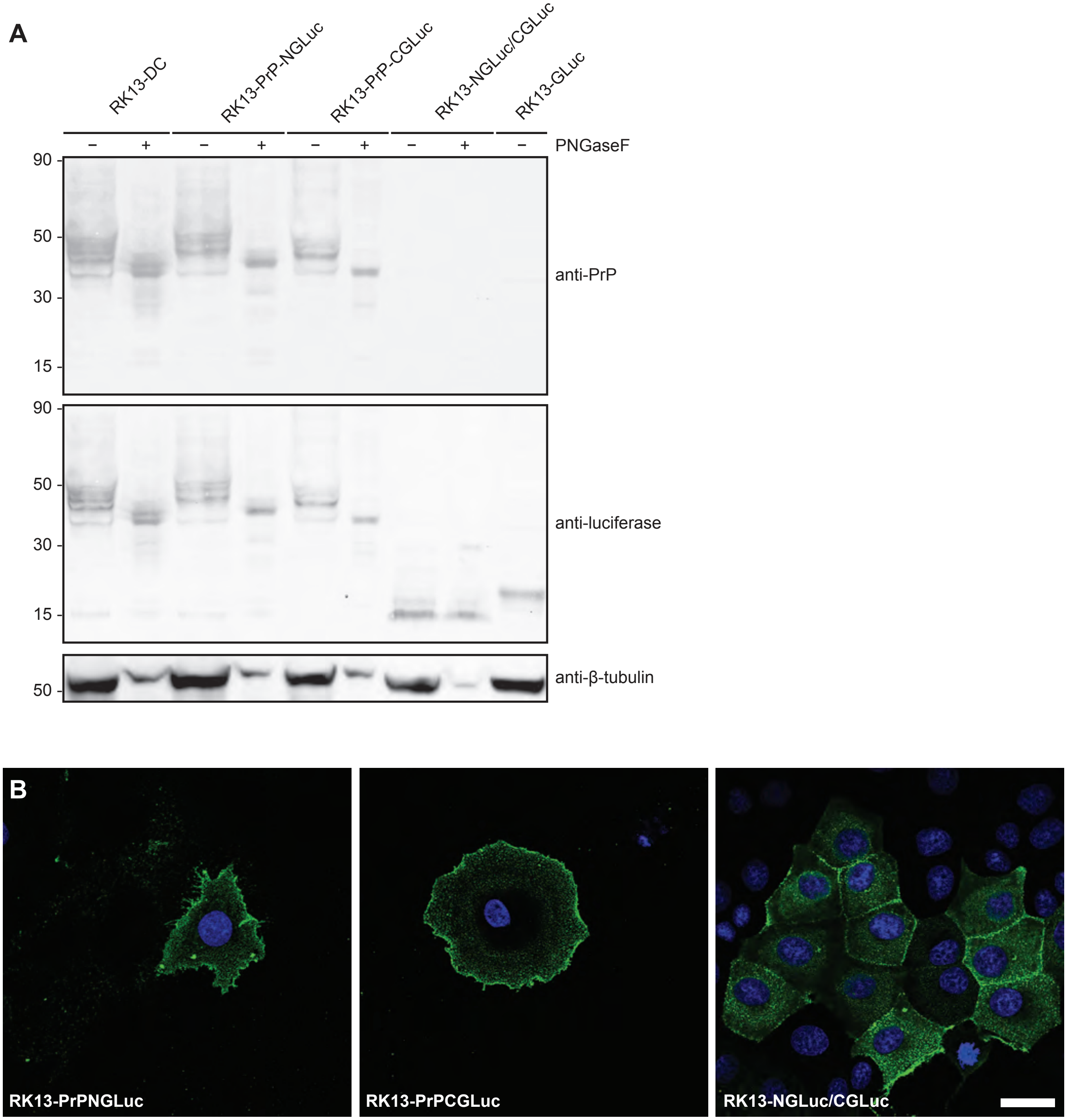
A
RK13-DC
RK13-PrP-NGLuc
RK13-PrP-CGLuc
RK13-NGLuc/CGLuc
RK13-GLuc
PNGaseF
90
50
30
15
anti-PrP
anti-luciferase
anti-β-tubulin
B
RK13-PrPNGLuc
RK13-PrPCGLuc
RK13-NGLuc/CGLuc

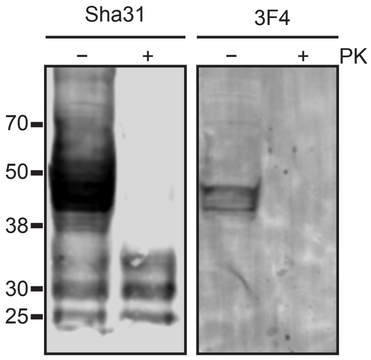

Sha31
3F4
− + − + PK
70
50
38
30
25

JTC-801
CAS: 244218-51-7
MW: 411.51
MF: $C_{26}H_{25}N_3O_2$

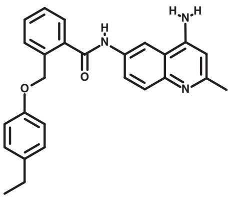

AEE788 (NVP-AEE788)
CAS: 497839-62-0
MW: 440.58
MF: $C_{27}H_{32}N_6$

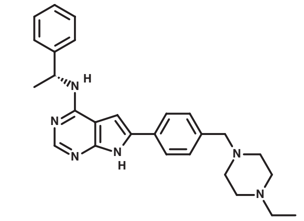

Idebenone
CAS: 58186-27-9
MW: 338.44
MF: $C_{19}H_{30}O_5$

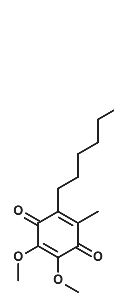

Etavirine (TMC125)
CAS: 269055-15-4
MW: 435.28
MF: $C_{20}H_{15}BrN_6O$

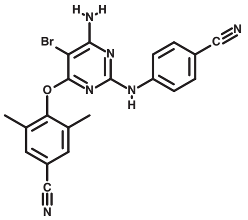

Ponatinib (AP24534)
CAS: 943319-70-8
MW: 532.56
MF: $C_{29}H_{27}F_3N_6O$

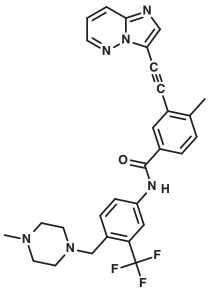

Cobicistat (GC-9350)
CAS: 1004316-88-4
MW: 776.02
MF: $C_{40}H_{53}N_7O_5S_2$

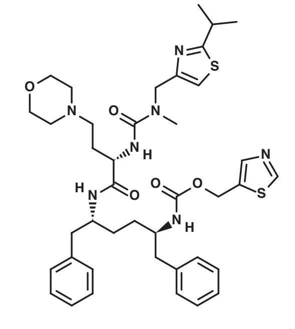

Flavopiridol (Alvocidib) HCl
CAS: 131740-09-5
MW: 438.30
MF: $C_{21}H_{21}Cl_2NO_5$

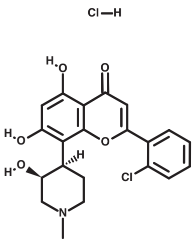

R406
CAS: 841290-80-0
MW: 470.45
MF: $C_{22}H_{23}FN_6O_5$

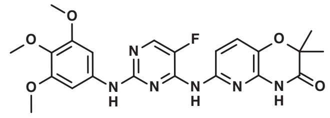

17-DMAG HCl (Alvespimycin)
CAS: 467214-21-7
MW: 653.21
MF: $C_{32}H_{49}ClN_4O_8$

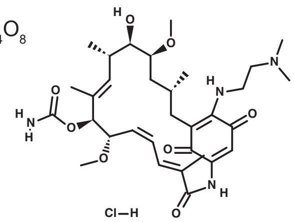

AZD5363
CAS: 1143532-39-1
MW: 428.92
MF: $C_{21}H_{25}ClN_6O_2$

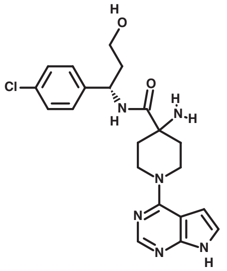

Azalomycin-B (Elaiophylin)
CAS: 37318-06-2
MW: 1025.27
MF. $C_{54}H_{88}O_{18}$

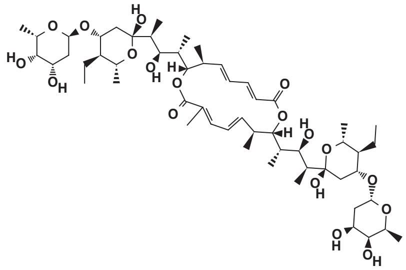

AST-1306
CAS: 1050500-29-2
MW: 621.08
MF: $C_{31}H_{26}ClFN_4O_5S$

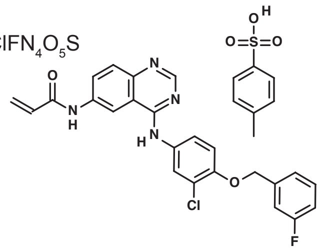

Dapivirine
CAS: 244767-67-7
MW: 329.40
MF: $C_{20}H_{19}N_5$

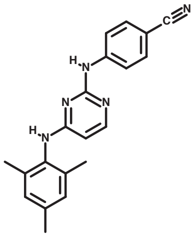

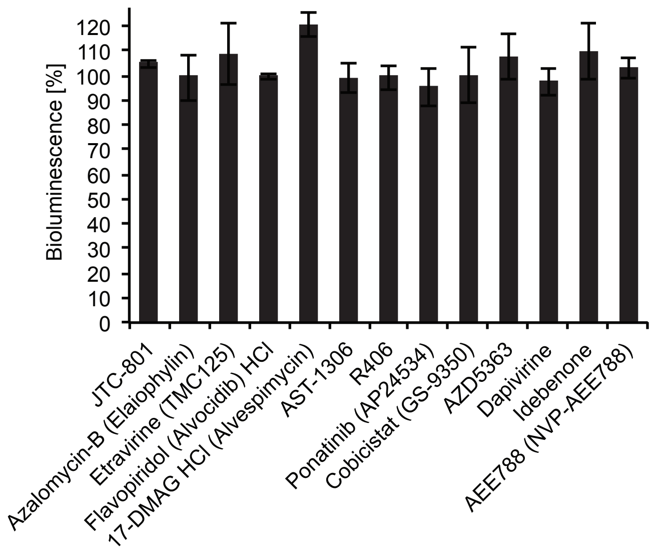

Bioluminescence [%]
120
110
100
90
80
70
60
50
40
30
20
10
0
JTC-801
Azalomycin-B (Elaiophylin)
Etravirine (TMC125)
Flavopiridol (Alvocidib) HCl
17-DMAG HCl (Alvespimycin)
AST-1306
R406
Ponatinib (AP24534)
Cobicistat (GS-9350)
AZD5363
Dapivirine
Idebenone
AEE788 (NVP-AEE788)

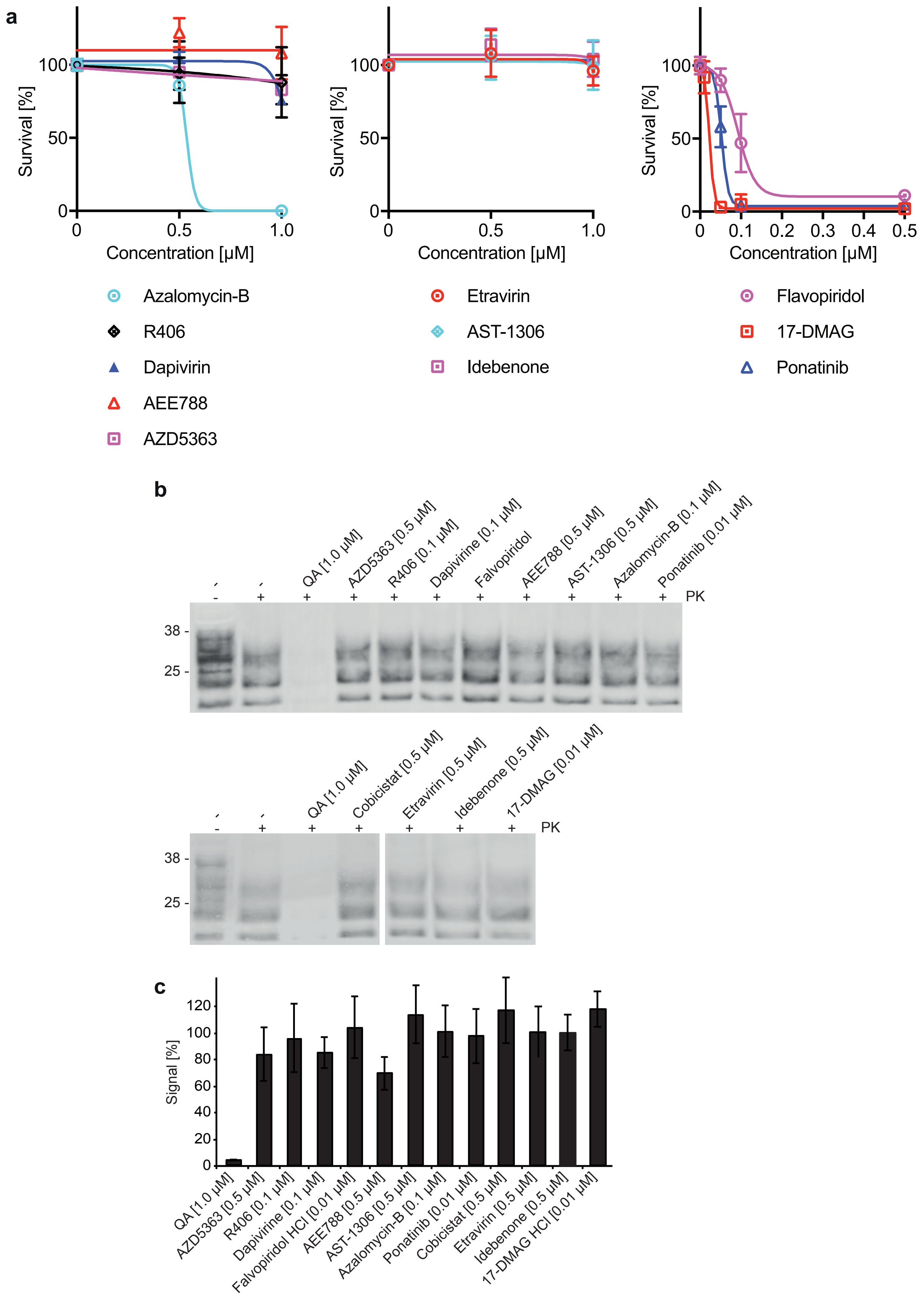
a
Survival [%]
100
50
0
0.5
1.0
Concentration [μM]
0.1
0.2
0.3
0.4
Azalomycin-B
R406
Dapivirin
AEE788
AZD5363
Etravirin
AST-1306
Idebenone
Flavopiridol
17-DMAG
Ponatinib
b
QA [1.0 μM]
AZD5363 [0.5 μM]
R406 [0.1 μM]
Dapivirine [0.1 μM]
Falvopiridol
AEE788 [0.5 μM]
AST-1306 [0.5 μM]
Azalomycin-B [0.1 μM]
Ponatinib [0.01 μM]
PK
38 -
25 -
Cobicistat [0.5 μM]
Etravirin [0.5 μM]
Idebenone [0.5 μM]
17-DMAG [0.01 μM]
c
Signal [%]
120
100
80
60
40
20
0
QA [1.0 μM]
AZD5363 [0.5 μM]
R406 [0.1 μM]
Dapivirine [0.1 μM]
Falvopiridol HCl [0.01 μM]
AEE788 [0.5 μM]
AST-1306 [0.5 μM]
Azalomycin-B [0.1 μM]
Ponatinib [0.01 μM]
Cobicistat [0.5 μM]
Etravirin [0.5 μM]
Idebenone [0.5 μM]
17-DMAG HCl [0.01 μM]

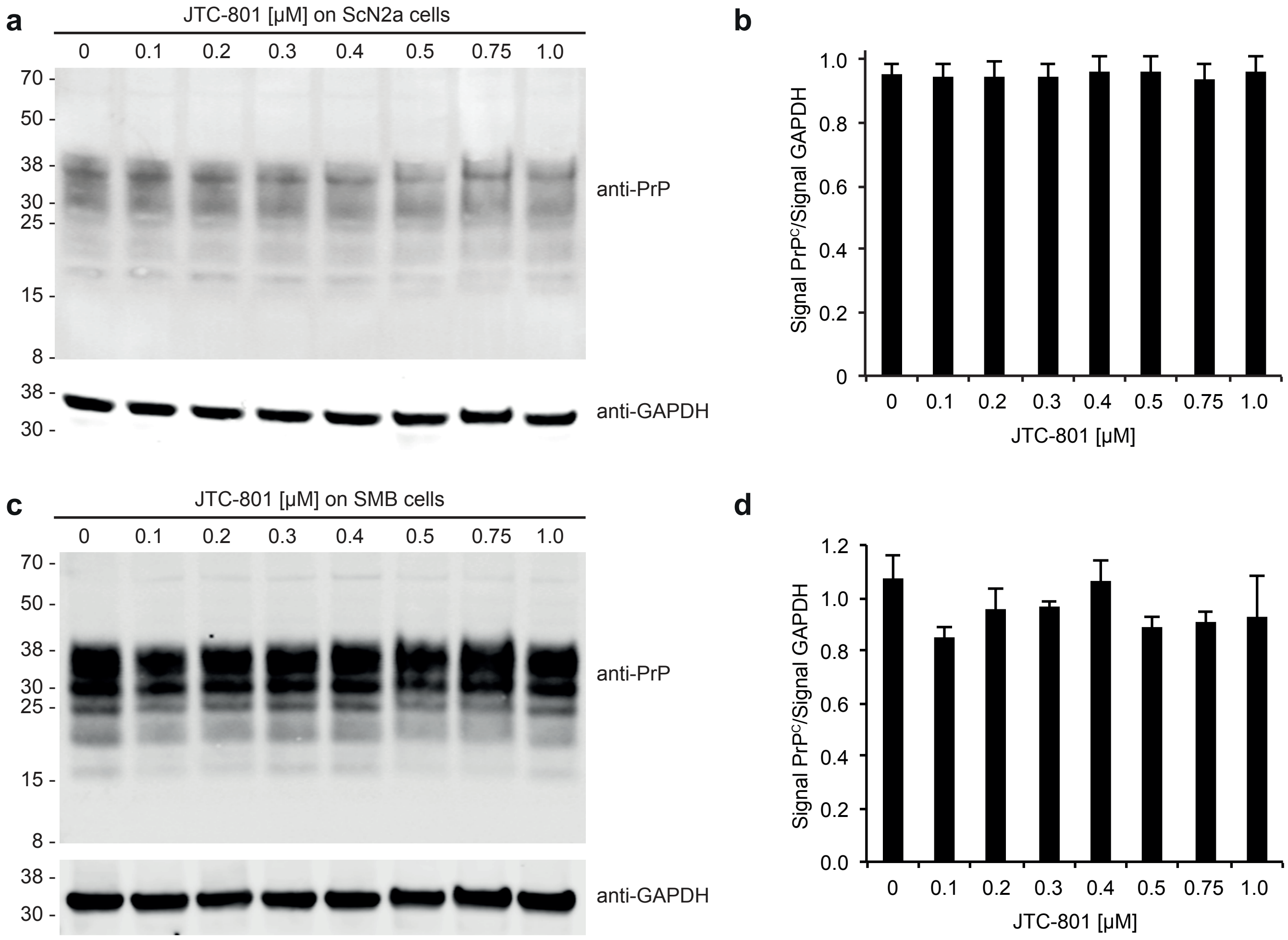
a
JTC-801 [µM] on ScN2a cells
0 0.1 0.2 0.3 0.4 0.5 0.75 1.0
70 50 38 30 25 15 8
anti-PrP
38 30
anti-GAPDH
b
Signal PrPC/Signal GAPDH
1.0 0.8 0.6 0.4 0.2 0
0 0.1 0.2 0.3 0.4 0.5 0.75 1.0
JTC-801 [µM]
c
JTC-801 [µM] on SMB cells
0 0.1 0.2 0.3 0.4 0.5 0.75 1.0
70 50 38 30 25 15 8
anti-PrP
38 30
anti-GAPDH
d
Signal PrPC/Signal GAPDH
1.2 1.0 0.8 0.6 0.4 0.2 0.0
0 0.1 0.2 0.3 0.4 0.5 0.75 1.0
JTC-801 [µM]
